# Evidence of Hypoxic Glial Cells in a Model of Ocular Hypertension

**DOI:** 10.1167/iovs.18-24977

**Published:** 2019-01

**Authors:** Assraa H. Jassim, Denise M. Inman

**Affiliations:** Department of Pharmaceutical Sciences, Northeast Ohio Medical University, Rootstown, Ohio, United States

**Keywords:** glutathione, hypoxia-inducible factor, optic neuropathy, Müller glia, astrocytes

## Abstract

**Purpose:**

Reoxygenation after hypoxia can increase reactive oxygen species and upregulate autophagy. We determined, for the first time, the impact of elevated IOP on hypoxia induction, superoxide accumulation, and autophagy in a bead model of glaucoma.

**Method:**

Ocular hypertension was achieved with magnetic bead injection into the anterior chamber. Before mice were killed, they were injected with pimonidazole for hypoxia detection and dihydroethidium (DHE) for superoxide detection. Total retinal ganglion cells (RGCs) and optic nerve (ON) axons were quantified, total glutathione (GSH) was measured, and retinal and ON protein and mRNA were analyzed for hypoxia (Hif-1α and Hif-2α), autophagy (LC3 and p62), and SOD2.

**Results:**

With IOP elevation (*P* < 0.0001), the retina showed significantly (*P* < 0.001) decreased GSH compared with control, and a significant decrease (*P* < 0.01) in RGC density compared with control. Pimonidazole-positive Müller glia, microglia, astrocytes, and RGCs were present in the retinas after 4 weeks of ocular hypertension but absent in both the control and after only 2 weeks of ocular hypertension. The ON showed significant axon degeneration (*P* < 0.0001). The mean intensity of DHE in the ganglion cell layer and ON significantly increased (*P* < 0.0001). The ratio of LC3-II to LC3-I revealed a significant increase (*P* < 0.05) in autophagic activity in hypertensive retinas compared with control.

**Conclusions:**

We report a novel observation of hypoxia and a significant decrease in GSH, likely contributing to superoxide accumulation, in the retinas of ocular hypertensive mice. The significant increase in the ratio of LC3-II to LC3-I suggests autophagy induction.

Glaucoma is a neurodegenerative disease that affects the function of retinal ganglion cells (RGCs) in transmitting visual information from the eye to the brain. Glaucoma is an optic neuropathy,^1^ a progressive and irreversible atrophy of the optic nerve (ON), and eventually RGC degeneration in the retina.^2^ In some models of glaucoma, IOP elevation initiates hypoxia that results in RGC axon degeneration.^3^ Hypoxia and oxidative stress are classic inducers of autophagy, the clearance of damaged macromolecules.^4^ Therefore, interaction among hypoxia, oxidative stress, and autophagy merits investigation for its possible role in the pathogenesis of glaucoma.

Oxidative stress results from an overproduction of reactive oxygen species (ROS) in the mitochondria.^5^ Although ROS have been viewed as damaging to the cell, controlled levels of ROS have a vital role in cell signaling.^6^ Superoxide, for example, generated during hypoxia, is both necessary and sufficient to activate the hypoxia-inducible factors-α (HIF-α) that are important for cellular adaptation to hypoxic stress.^7^–^10^

Hypoxia and the subsequent reoxygenation exposes cells to severe stress in the retina, optic nerve head (ONH), and optic nerve (ON).^3^,^11^ Hypoxia causes genetic instability,^12^ activates autophagy,^13^,^14^ regulates protein translation and degradation, and, together with ROS generated by reoxygenation, causes protein damage and misfolding.^15^ During hypoxia, cells initiate a variety of defense responses mediated by the HIFs, heterodimer transcription factors consisting of a stable, oxygen-insensitive HIF-β subunit and one of the three oxygen-sensitive HIF-α subunits (HIF-1α, HIF-2α, or HIF-3α) that together bind to the hypoxia response element in the nucleus to promote the transcription of hypoxia-responsive genes, including erythropoietin and VEGF.^16^,^17^ HIF-α enables metabolic adaptation to hypoxia through increased glycolysis through the upregulation of glucose transporters.^18^ The pathophysiological role of hypoxic signaling in glaucomatous neurodegeneration was suggested by immunostaining for HIF-1 in human glaucomatous retina from patients who exhibited visual function deficit.^11^

Autophagy, a catabolic digestion process of cellular macromolecules and organelles, plays an important role in protecting cells against adverse conditions such as oxidative stress, hypoxia, and starvation; events observed during glaucoma.^19^ The process makes use of cargo receptors, such as microtubule-associated protein 1A/1B-light chain 3 (LC3) and SQSTM1/p62, recognizing ubiquitinated cellular waste and initiating its engulfment by the autophagosome. The autophagosome eventually fuses with the lysosome where the lysosomal proteases degrade the cargo. By eliminating damaged proteins and organelles, autophagy could have a cytoprotective role in neurodegeneration.^19^

Previous research has reported hypoxia in glaucoma; however, no one has yet shown hypoxic glia in the retina, nor the interaction among hypoxia, oxidative stress, and autophagy while using the bead glaucoma model. Additionally, we examined antioxidants and oxidative stress in a hypoxic context. We observed hypoxia in the ocular hypertensive retina in RGCs, Müller glia, microglia, and astrocytes. Hypoxia was also accompanied by an accumulation of superoxide and downregulation of the antioxidant glutathione (GSH), circumstances that may contribute to the progression of glaucoma.

## Materials and Methods

### Animals

Both sexes of C57BL/6J (B6) mice of roughly 3 months of age were used. All mice were originally obtained from Jackson Laboratories (Bar Harbor, ME, USA), then bred and housed in a specific pathogen-free barrier facility on a 12-hour light-dark cycle at the Northeast Ohio Medical University (Rootstown, OH, USA). Supplementary Table S1 shows the number of mouse samples used in all experiments. All procedures were approved by the Institutional Animal Care and Use Committee and performed in accordance with the ARVO Statement for the Use of Animals in Ophthalmic and Vision Research.

### Glaucoma Model

**Figure 1:**
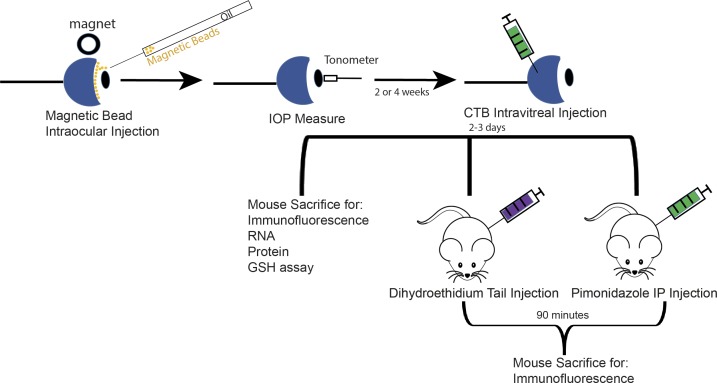
Experimental design. OHT was induced by magnetic bead intracameral injection. IOP was measured before OHT (baseline), day 1 after induction, then once weekly for 2 or 4 weeks. CTB intravitreal injection was performed 2 to 3 days before animals were killed to determine integrity of anterograde axon transport. One group of mice received an IP injection of 60 mg/kg pimonidazole, then was killed 90 minutes later, to assess hypoxia at 2 and 4 weeks after OHT. A separate group of mice received a tail vein injection of DHE, then was killed 90 minutes later to determine superoxide level 4 weeks after OHT. Separate groups of mice were killed for protein, RNA, and GSH analysis. Sample sizes are listed in Supplementary Table S1.

Ocular hypertension was mechanically induced in 2.5% isoflurane-anesthetized B6 mice, using 1 μL of magnetic microbeads (COMPEL COOH-Modified 8-μm diameter, UMC4001; Bangs Laboratories, Fishers, IN, USA) injected into the anterior chamber of the eye using a glass-pulled micropipette connected to a manual microsyringe pump (World Precision Instruments, Sarasota, FL, USA). Magnetic beads were distributed along the trabecular meshwork using a neodymium magnet that draws them into the iridocorneal angle. The microbeads block the aqueous humor outflow pathway through the trabecular meshwork; the accumulation of aqueous humor causes an acute increase in IOP.^20^–^22^ Both eyes were injected with beads to eliminate the confounding factor of contralateral eye effects on glial activation.^23^ Separate B6 mice served as controls. This model provided reliability and minimum damage to ocular structures. See Figure 1 for the experimental design.

### IOP Measurements

Ten IOP measurements per eye of lightly isoflurane-anesthetized mice (2.5%) were taken and averaged; a baseline measurement was taken before bead injection, then weekly measurements after bead injection for 2 or 4 weeks using the TonoLab tonometer (iCare Finland Oy, Vantaa, Finland). IOP integral (mm Hg-days exposure over baseline) was also calculated.^24^

### Anterograde Transport Evaluation

All materials and reagents were obtained from ThermoFisher Scientific (Waltham, MA, USA) unless otherwise indicated. Two to 3 days before euthanizing mice, anterograde labeling of RGCs was performed by injecting the vitreal chamber of the eye of anesthetized mice with 1.5 μL of the neuroanatomical tracer cholera toxin B-subunit (CTB; C22841). CTB is conjugated to the photo-stable, bright, and pH-insensitive Alexa Fluor-488 and is transported anterogradely along RGC axons after endocytosis of GM1, the CTB receptor. After perfusion (see below), the superior colliculus (SC), the primary and most distal site of the RGC projection in rodents, was coronally sectioned at 50 μm using a Leica (Wetzlar, Germany) freezing microtome, then 10 representative SC sections were mounted on slides, imaged using Zeiss AxioZoom V16 (AxioCam MRm Rev.3; Zeiss, Jena, Germany), and analyzed using a custom-written macro for ImageJ^25^ (http://imagej.nih.gov/ij/; provided in the public domain by the National Institutes of Health, Bethesda, MD, USA) to calculate percentage area fraction of CTB.^26^

### Hypoxia Detection and Immunofluorescence

To detect cellular hypoxia, 60 mg/kg pimonidazole hydrochloride (Hypoxyprobe Green kit; Hypoxyprobe, Burlington, MA, USA) diluted in sterile PBS was administered by intraperitoneal (IP) injection 90 minutes before animals were killed. Pimonidazole forms covalent adducts in cells that have a partial pressure of oxygen less than 10 mm Hg. The subsequent staining of tissue sections with an anti-pimonidazole antibody reveals the presence of these hypoxic cells. After perfusion with 1% paraformaldehyde (PFA), retinas attached to ONs were post fixed in 4% PFA for 30 minutes. Retinas were cryosectioned, then sections were incubated for 1 hour in blocking agent (1% Triton X-100, 0.5% BSA, 0.9% NaCl, and 5% donkey serum [DKS; Jackson ImmunoResearch, West Grove, PA, USA] in 1% PBS; PBS-T-BSA). Slides were incubated with anti-pimonidazole antibody (FITC-conjugated mouse anti-pimonidazole, 1:200) in blocking solution overnight. The next day, retinal and ON sections were rinsed in PBS and cover-slipped for confocal imaging using Leica application Suite X 3.1.1.15751 (Leica Microsystems, Buffalo Grove, IL, USA).^3^,^27^ Six slides (4 sections/slide) were imaged for the 2-week time point, and 10 to 12 slides (four sections/slide) were imaged for the 4-week time point. To confirm the detection of hypoxia using pimonidazole, primary antibody against pimonidazole conjugated to FITC (1:200) was added to ON and retinal sections from mice that had received no pimonidazole IP injection; no florescence was detected.

### Tail Vein Injection of Dihydroethidium

To detect superoxide accumulation, dihydroethidium (DHE; Sigma-Aldrich, St. Louis, MO, USA) was injected by tail vein into control and bead-injected mice (0.2 mg DHE per mouse). DHE is oxidized to red fluorescent ethidium by superoxide to indicate intracellular superoxide levels. Ninety minutes after DHE injection, mice were perfused with 4% PFA, the retinal and ON tissue was cryoprotected, embedded, and sectioned, then observed with a Leica confocal microscope at 570 nm. Intensity analysis of DHE in longitudinal ON sections and sagittal retinal sections was measured using ImageJ.

### Perfusion and Tissue Preparation

Mice were killed at the end of weeks 2 and 4 after bead injection with an overdose of sodium pentobarbital (Beuthanasia-D, 390 mg/kg, IP; Merck Animal Health, Baton Rouge, LA, USA) then perfused transcardially with 0.1M PBS, then with 1% or 4% PFA. Retina, ON, and SC were harvested for histology, immunofluorescence, quantitative PCR, glutathione assay, and Simple Western, as previously described.^24^,^28^,^29^

### Cryosectioning

Fixed retinas and ONs were cryoprotected in 30% sucrose and 0.02% sodium azide in 0.1M PBS and embedded in optimal cutting temperature medium for sagittal sectioning (retinas) and longitudinal sectioning (ONs) at 10 to 15 μm using a Leica cryostat.

### Immunofluorescence (IF)

Eyes were freshly dissected after transcardial perfusion with 0.1M PBS, immersion fixed in 4% PFA for 15 to 30 minutes, then whole-mount retinas were dissected for RGC labeling with RNA binding protein with multiple splicing (RBPMS), as previously described.^30^ ONs attached to the brain were dissected from the skull, then post fixed for 24 hours in 4% PFA. The following antibodies were used: RBPMS (an RGC marker, 1:200, GTX118619; GeneTex, Irvine, CA, USA), glial fibrillary acidic protein (GFAP, 1:500, ab53554; Abcam, Cambridge, MA, USA), ionized calcium binding adaptor molecule 1 (Iba-1, a microglia marker, 1:200, 019-10741; Wako, Richmond, VA, USA), neurofilament 200 (NF200, 1:100, N4142; Sigma-Aldrich), p62 (sequestosome 1 [SQSTM1], 1:100, ab56416; Abcam), HIF-1α (1:100, sc-13515; Santa Cruz Biotechnology, Dallas, TX, USA), HIF-2α (1:100, sc-13596; Santa Cruz Biotechnology), and superoxide dismutase 2 (SOD2, 1:100, sc-133134; Santa Cruz Biotechnology). The above dilutions of primary antibodies were determined by optimization with different serial dilutions. Whole mounts and sections were rinsed in PBS, blocked with 5% DKS, and 1% Triton X-100 in 1X PBS, quenched using 0.3% H_2_O_2_, then incubated with primary antibodies prepared in PBS-T-BSA for 2 days (whole mounts) or overnight (sections) at 4°C. Tissue and sections were rinsed in PBS then blocked in PBS-T-BSA. Secondary antibodies (1:250; Alexa- Fluor488, 594, or 647; Jackson ImmunoResearch) prepared in PBS-T-BSA were added and incubated overnight at 4°C (whole mount) or for 2 hours (sections). DAPI (4′,6-diamidino-2-phenylindole; 1:2000) was applied, then rinsed before sections were cover-slipped using Fluoromount-G (SouthernBiotech, Birmingham, AL, USA). Six to 10 representative slides (four sections/slide) were imaged. To confirm specificity, we incubated sections with secondary antibodies but without primary antibodies. Whole mounts were mounted nerve fiber layer (NFL) side up on slides, then cover-slipped. Images were captured using a Leica DMi8 confocal microscope integrated with Leica confocal microscope (mentioned above) or Olympus FV-1000 confocal microscope (Olympus, Cedar Valley, PA, USA). Mean intensity of p62 and SOD2 in the regions of interest were analyzed using four sections per slide, and three slides per retina or ON using Image J.

### Protein Extraction

Retinal and myelinated ON proteins were collected into tubes containing T-PER buffer with HALT protease and phosphatase inhibitors, then disrupted with a Branson Sonifier (3-second pulse at 10% amplitude) to create a protein lysate. Total protein concentration was measured by Bicinchoninic Acid (BCA) assay kit.

### Capillary-Based Electrophoresis for Protein Quantification

Proteins were analyzed by capillary tube–based electrophoresis immunoassay using the Wes, a Protein Simple instrument that separates proteins by electrical charge in capillary tubes and allows binding of primary antibody then protein visualization within the capillary.^29^ The Wes allows us to assay individual ONs and quantify the amount of specific proteins within a lysate. Wes results are normalized to total protein in the sample, as determined by capillary electrophoresis. Protein analysis for p62 and SOD-2 was repeated at least three times with biological replicates.

### Western Blot Analysis

Proteins were separated on denaturing polyacrylamide gels and transferred by electrophoresis to a polyvinyl difluoride membrane. The addition of a phosphatidylethanolamine moiety to Light Chain 3 (LC3b-I) during autophagosome formation causes LC3b-II to migrate on SDS-PAGE, as if it is smaller than LC3b-I, a circumstance best detected using PAGE. Therefore, we analyzed LC3-I and -II in 20 μg retina using Western blot. Blots were probed with β-actin, and LC3 A/B primary antibodies (1:1000, 4108; Cell Signaling Technologies, Danvers, MA, USA). In addition, we analyzed HIF-1α and HIF-2α in 50 μg retina after 2 weeks and 4 weeks of ocular hypertension. Appropriate secondary antibodies conjugated to horseradish peroxidase were used, then developed with chemiluminescence (Pierce, Rockford, IL, USA). Band density was measured by using the lane profile analysis function in AlphaView SA version 3.4.0 software and normalized to β-actin within lane band density.^28^

### Glutathione Assay

Fresh dissected retinas and ONs were prepared according to the Glutathione Assay Kit instructions (703002; Cayman Chemical, Ann Arbor, MI, USA) and as previously described.^24^ GSH is an antioxidant scavenger that reacts with Ellman's reagent, producing a disulfide that is reduced by glutathione reductase. Measurement of absorbance of the disulfide at 405 nm using a plate reader (Spectra Max M5, softmax pro 6.5.1; Molecular Devices, Sunnyvale, CA, USA) provided an accurate estimate of total GSH. Total GSH was normalized to the total protein in the sample as measured by BCA Assay.

### Quantitative Real-time PCR (qPCR)

Total RNA was isolated from fresh retinas and ONs using TriZol.^24^ First-strand cDNA synthesis was performed using the Verso cDNA synthesis kit before qPCR using the Quant Studio 6 Flex System instrument (ThermoFisher Scientific, Waltham, MA, USA). The following TaqMan assays were used to assess gene expression*: Hif-1a*, *Hif-2a*, *P62*, *Glut-1*. Three housekeeping genes (*Hprt*, *β-actin*, *Rpl 13a*) were also run. Retinas and ONs were normalized to *β-actin*, as it was the most reliable housekeeping gene across all samples.

### Histopathology for Light Microscopy

ONs were post fixed in 2% PFA + 2% glutaraldehyde in 0.1 M sodium cacodylate buffer (CB; pH 7.4) for 48 hours. Tissue was processed as previously described.^28^,^31^ Briefly, after washing three times with CB, tissues were incubated in 2% osmium tetroxide in CB for 45 minutes. Dehydration of tissue through a graded series of alcohols followed by propylene oxide, then increasing concentrations of PolyBed resin culminated in final embedding and polymerization in PolyBed resin (Araldite 502/Polybed 812 kit; Polysciences, Inc, Warrington, PA, USA). Using a Leica ultramicrotome, sections (500 nm) were cut, mounted on slides, and then stained with 1% ρ-phenylenediamine (PPD).

### Quantification of RGCs and Axons

Unbiased stereological analysis of RBPMS-positive RGCs in whole-mount retina using a ×40 objective or axons in PPD-stained ON using a ×100 objective was performed as previously described using the optical fractionator module within StereoInvestigator (MicroBrightfield Bioscience, Williston, VT, USA).^24^ A 50 × 50-μm or 5 × 5-μm counting frame (retina and ON, respectively) was used across approximately 40 sites (10%) for both tissue types. The coefficient of error (Schmitz-Hof) was maintained at 0.05 or below, ensuring sufficient sampling rate.

### Statistical Analysis

Statistical analysis was performed using GraphPad Prism 7 (GraphPad, La Jolla, CA, USA). Data were expressed as mean ± SEM and analyzed using unpaired, two-tailed Student's *t*-test (two groups) when comparing control versus experimental and 1-way ANOVA (three groups) followed by Tukey's multiple-comparison post hoc test when making comparisons across more than two groups. A *P* value <0.05 was considered statistically significant.

## Results

### Degeneration of RGC Soma and Axons and Impaired Anterograde Transport

**Figure 2:**
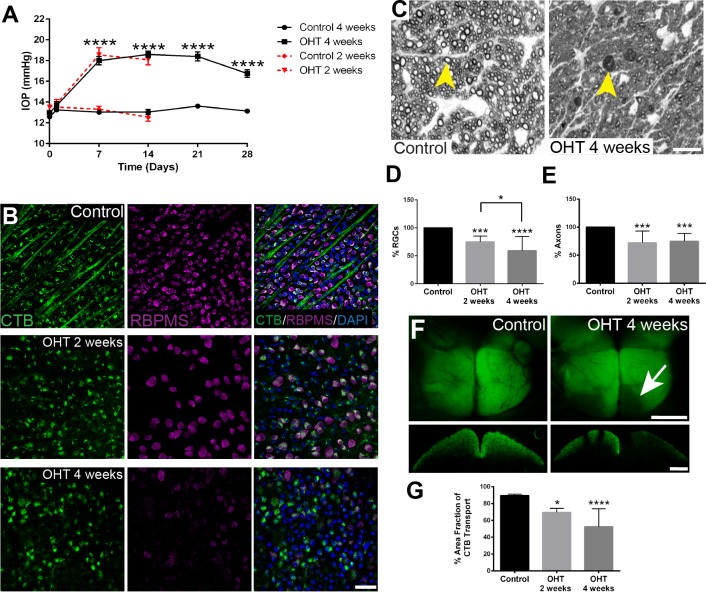
Degeneration of RGC somata and axons and impaired anterograde transport after 2 and 4 weeks of OHT. (A) Significant IOP elevation was observed after 2 (control [Ctrl]: n = 16, OHT: n = 17) and 4 weeks (Ctrl: n = 29, OHT: n = 54) of OHT versus control. IOP elevation was significantly higher than baseline from day 7 through 28 (****P < 0.0001). An average of 10 IOP measurements were taken per eye, per time point. (B) Immunofluorescence of RGCs (RBPMS, magenta) in whole-mount OHT (2 and 4 weeks) compared with control retinas. RGCs also are labeled with CTB (green). Scale bar: 20 μm. (C) ON cross sections with an arrowhead pointing to a healthy axon (right) in a control ON; in the ON taken from a mouse with OHT (left), the arrowhead points to a degenerating axon. Scale bar: 50 μm. (D) RBPMS-positive RGC somata in whole-mount retinas were quantified 14 and 28 days after OHT using unbiased stereology. There were significantly fewer (F_2,53_ = 29.76, ****P < 0.0001) RGCs after 2 weeks (n = 11) and after 4 weeks (n = 25) of OHT compared with control retina (n = 20). Tukey's post hoc test showed a significant decrease in RGCs after 2 (**P < 0.01) and 4 (****P < 0.0001) weeks of OHT compared with control, and after 4 weeks of OHT compared with 2 weeks (*P < 0.05). RGC density in Control = 4143 ± 224.6; after 2 weeks of OHT = 3526 ± 147.2; and after 4 weeks of OHT = 1925 ± 167. (E) Significant axon loss at 2 weeks (n = 9) and 4 weeks (n = 12) after OHT (F_2,30_ = 13.96, ***P < 0.001) was observed in ONs compared with control (n = 12). Tukey's post hoc test showed significant axon loss (***P < 0.001) after 2 weeks and 4 weeks of OHT compared with control. No significant difference in axon loss was observed between 2 and 4 weeks after OHT. Axon number in Control = 48,040 ± 3766; after 2 weeks of OHT = 31,062 ± 4180; and after 4 weeks of OHT = 24,253 ± 2269. (F) Dorsal views (Scale bar: 1000 μm) and coronal sections (Scale bar: 500 μm) of the SC show significant anterograde axonal transport deficit. Arrow shows lack of CTB label (green) in the lower left quadrant of each lobe compared with the control. (G) Percent area fraction of CTB in the SC of OHT mice was significantly lower (F_2,27_ = 17.35, ****P < 0.0001) at 2 weeks (n = 8) and 4 weeks (n = 10) after OHT compared with control (n = 12). Tukey's post hoc test showed a significant decrease in percent area fraction of CTB between 2 (*P < 0.05) and 4 (****P < 0.0001) weeks after OHT compared with control. No significant difference in percent area fraction was observed between 2 weeks and 4 weeks after OHT.

We measured IOP, quantified RGCs and their axons, and assessed anterograde axon transport to the SC in bead-injected mouse eyes and brain to demonstrate the impact of ocular hypertension (OHT). A significant IOP increase was observed in bead-injected mice 1 week after injection; significant elevation over control occurred for the 2-week and 4-week OHT groups (Fig. 2A). IOP integral was significantly higher (*P* < 0.0001) in OHT mice after 2 weeks (100.5 ± 10.7) compared with control mice (8.28 ± 3.7). IOP integral was also significantly higher (*P* < 0.0001) in 4-week OHT mice (152.2 ± 7.4) compared with control (17.79 ± 7.11). Two weeks after OHT, we observed a significant decrease in RGC density, and a further decrease was observed 4 weeks after OHT compared with control mice (Figs. 2B, 2D). Axon numbers as quantified in cross sections of ON were significantly decreased with OHT (2 and 4 weeks after injection) compared with control ON (Figs. 2C, 2E). CTB labeling in the SC, a reflection of anterograde axon transport, was significantly decreased at 2 and 4 weeks of OHT compared with control (Figs. 2F, 2G). Taken together, we concluded that the bead model effectively induced glaucoma progression 2 and 4 weeks after OHT.

### Detection of Hypoxic Glia and Neurons

**Figure 3:**
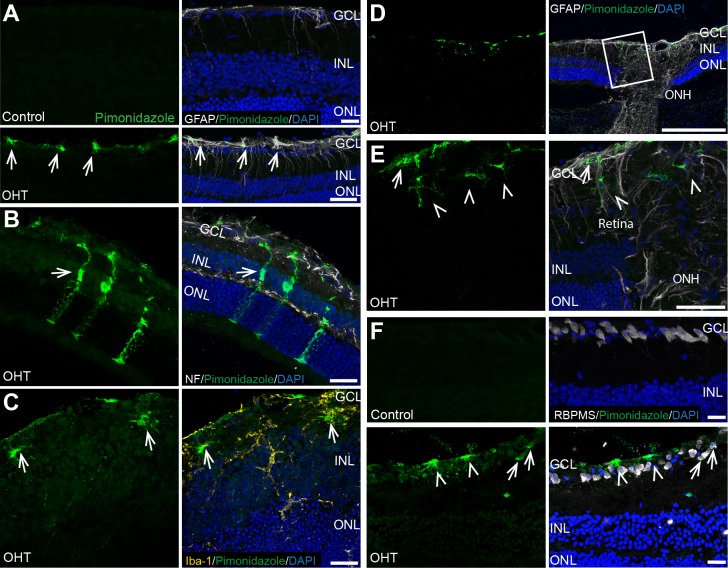
Detection of hypoxic glia and neurons in the retina, 4 weeks after OHT. (A) Sagittal section of control retina with no visible pimonidazole immunolabeling compared with OHT retina showing pimonidazole-positive astrocytes in the NFL, indicated by the arrows. GFAP (astrocyte labeling, white), pimonidazole (green), DAPI (cell nuclei, blue). Retinal layers labeled GCL, INL, and ONL. Scale bar: 25 μm. (B) OHT retina showing pimonidazole-positive Müller glia (arrow). Neurofilament (NF, white), pimonidazole (green), DAPI (cell nuclei, blue). Scale bar: 25 μm. (C) Sagittal section of OHT retina showing pimonidazole-positive microglia (arrows). Ionized calcium binding adaptor molecule 1 (Iba-1, yellow), pimonidazole (green), DAPI (cell nuclei, blue). Scale bar: 25 μm. (D) Sagittal section of OHT retina with visible ON and ONH. ONH shows no pimonidazole (green) adduct accumulation. Scale bar: 250 μm. (E) Inset from (D) sagittal section of OHT retina showing pimonidazole-positive (green) astrocytes (arrow) and microglia (arrowheads). Scale bar: 50 μm. (F) Sagittal section of control retina labeled with RBPMS (white) showing absence of hypoxia compared with OHT retina showing pimonidazole-positive RGCs (arrows) and astrocytes (arrowheads). RBPMS (RGC labeling, white), pimonidazole (hypoxia detection, green), and DAPI (blue). Scale bar: 20 μm, n = 10 independent OHT eyes and 8 independent control eyes.

To determine the level of hypoxia with OHT, we used IP injection of pimonidazole, a compound that generates an adduct with thiol-containing proteins in cells experiencing a partial pressure of oxygen below 10 mm Hg. Pimonidazole adduct labeling was detected in the NFL, ganglion cell layer (GCL), and the inner nuclear layer (INL) of bead-injected retina compared with control retina (Fig. 3). Hypoxia was detected after 4 weeks of OHT in Müller glia, microglia, and astrocytes more often than in RGCs within the retina (Figs. 3A–F). Of the 10 OHT retinas with pimonidazole-positive cells, all 10 had astrocytes that were pimonidazole-positive. Approximately three retinas each were observed to have pimonidazole-positive Müller glia, microglia, and RGCs. Pimonidazole-positive astrocytes were coincident with each of the other cell types within a retina, but the only other combinations were pimonidazole-positive RGCs and Müller glia that were observed in two retinas. Four weeks after OHT, the myelinated portion of the longitudinal ON showed no pimonidazole-positive axons (data not shown). No pimonidazole adduct labeling was detected in retina or the myelinated portion of the ON after 2 weeks of OHT (data not shown).

### Upregulation of *Hif-1α*, *Hif-2 α*, and *Glut-1* mRNA

**Figure 4:**
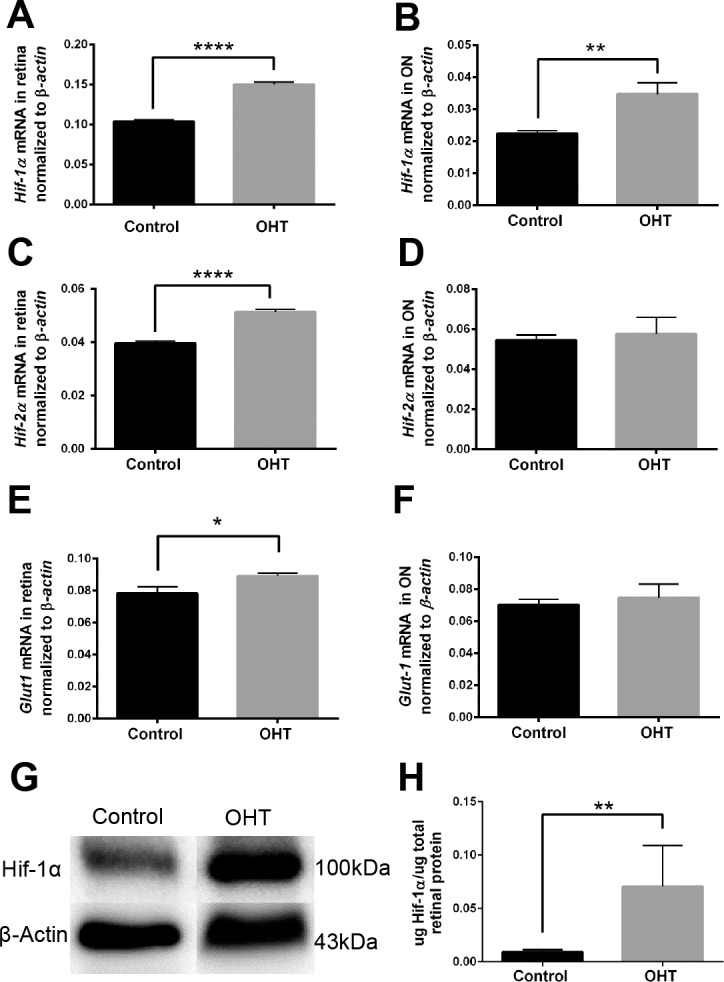
Upregulation of hypoxia transcripts and downstream target Glut-1, 4 weeks after OHT. (A) Hif-1α mRNA was significantly increased in OHT retinas (n = 3 in control, n = 4 in OHT; ****P < 0.0001). (B) Hif-1α mRNA was significantly increased in ONs with OHT (n = 3 in control, n = 7 in OHT; **P < 0.01). (C) Hif-2α mRNA was significantly increased in retinas with OHT (n = 3 control, n = 4 OHT; ****P < 0.0001). (D) There was no difference in Hif-2α mRNA in ON (n = 3 control, n = 7 OHT; P > 0.05). (E) Glut-1 expression was significantly increased in the OHT retinas (n = 3 control, n = 4 OHT; *P < 0.02). (F) There was no significant difference between control (n = 3) and OHT (n = 7), P > 0.05 for Glut-1 mRNA in ONs. All transcript expression normalized to β-actin. (G, H) A significant (**P < 0.01) increase of HIF-1α protein was observed in OHT retinas (n = 4) compared with control (n = 3).

After 4 weeks of OHT, hypoxia induction was also determined by detecting the significant increase in transcript expression of *Hif-1α* in OHT compared with control retina and ON (Figs. 4A, 4B). Interestingly, *Hif-2α* expression in retina was significantly increased in OHT compared with control (Fig. 4C); but in the ON, OHT (0.058 ± 0.008) was not different from control (0.054 ± 0.003, *P* > 0.05) (Fig. 4D). In keeping with the ability of *Hif-1α* to regulate glucose transporter expression, *Glut-1* transcript expression was significantly increased in OHT compared with control retina (Fig. 4E) but was not different from control in ON (Fig. 4F).

### HIF-1α and HIF-2α Protein

The level of HIF-1α significantly increased in OHT retina compared with control at 4 weeks (Figs. 4G, 4H). After 2 weeks of OHT, there was no significant difference between OHT and control retina in the level of either HIF-1α (0.146 ± 0.018, *n* = 4 OHT vs. 0.126 ± 0.014, *n* = 4 control) or HIF-2α (0.114 ± 0.020, *n* = 4 OHT vs. 0.135 ± 0.042, *n* = 3 control).

**Figure 5:**
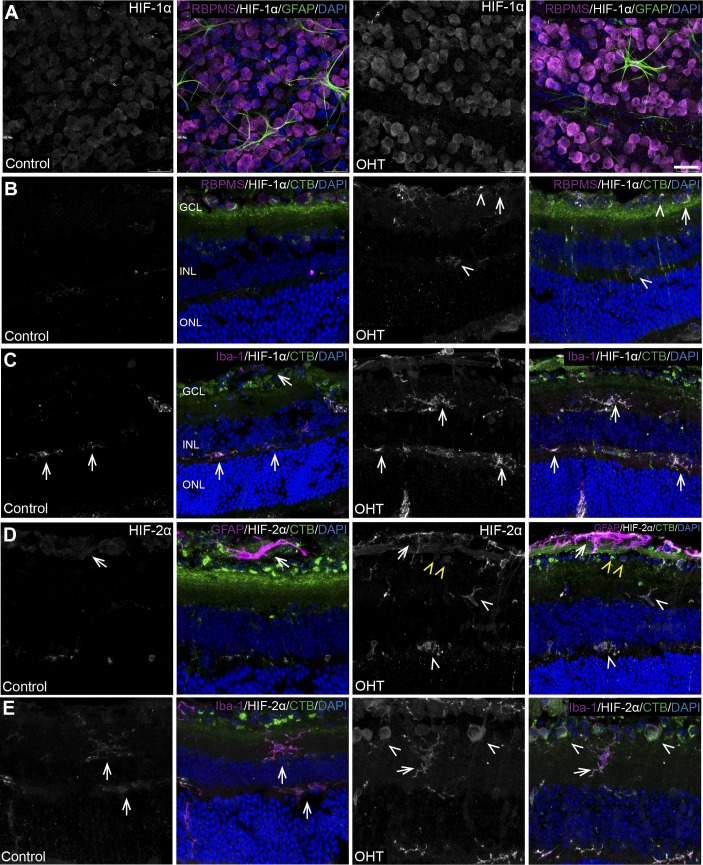
Significant increases of HIF-1α and HIF-2α, colocalized with GFAP and Iba-1 in retina after 4 weeks of OHT. (A) Whole-mount retina shows an increase in HIF-1α levels (white) with OHT compared with control. Colocalization of HIF-1α (white) with RBPMS (magenta) in RGCs. GFAP (green) and DAPI (blue). Ctrl: n = 4, OHT: n = 6. Scale bar: 25 μm. (B) Sagittal section of OHT retina shows an increase in HIF-1α level (white) compared with control. Colocalization of HIF-1α (white) with RBPMS (magenta) in RGCs (arrows) and in glia in the NFL and the INL (arrowheads). (C) Sagittal section of OHT retina shows an increase in HIF-1α level (white) compared with control. Colocalization of HIF-1α (white) with Iba-1 (magenta) in microglia (arrows) of retina. (D) Sagittal section of OHT retina shows an increase in HIF-2α level (white) compared with control. Colocalization of HIF-2α (white) with GFAP (magenta) in astrocytes in the NFL (arrows). HIF-2α localized to microglia in the inner and outer plexiform layers (white arrowheads) and in GCL neurons (yellow arrowheads). (E) Sagittal section of OHT retina shows an increase in HIF-2α level (white) compared with control. Colocalization of HIF-2α (white) with Iba-1 (magenta) in microglia (arrows). HIF-2α is also localized to RGCs in the GCL. CTB (green) and DAPI (blue). n = 3 independent control retinas and 3 independent OHT retinas. Scale bar: 25 μm.

Immunolabeling with antibodies against HIF-1α and HIF-2α showed an increase in OHT retina after 4 weeks compared with control. HIF-1α and HIF-2α colocalized with RBPMS or CTB-positive RGCs (Figs. 5A, 5B) as well as with GFAP (Fig. 5D) in the NFL, and with Iba-1 throughout the outer plexiform layer and the inner retina (Figs. 5C, 5E).

**Figure 6:**
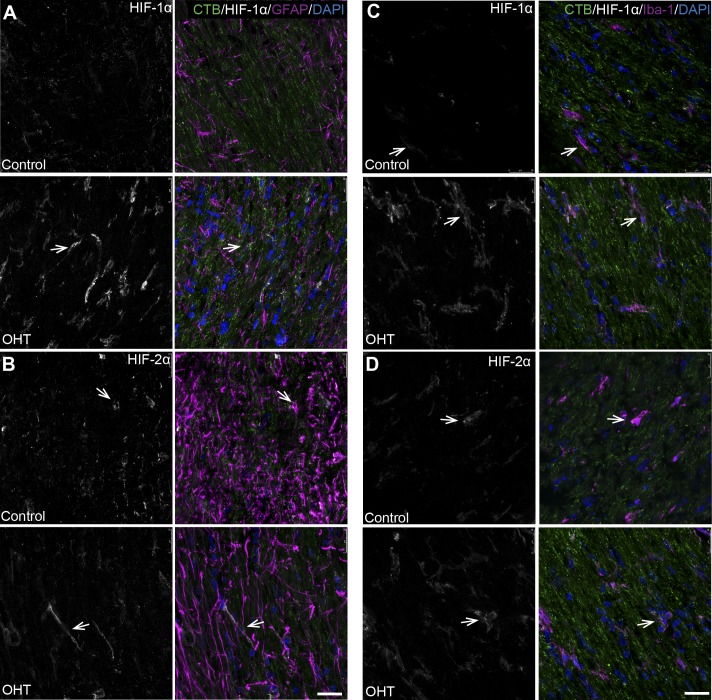
Colocalization of HIF-1α and HIF-2α with GFAP and Iba-1, with a significant increase of HIF-1α in ON after 4 weeks of OHT. Sections oriented so globe is superior. (A) Longitudinal section of OHT ON shows an increase in HIF-1α level (white) compared with control. Colocalization of HIF-1α (white) with GFAP (magenta) in astrocytes of OHT ON (arrows). (B) Longitudinal section of ON shows no difference in HIF-2α level (white) in OHT ON compared with control. Colocalization of HIF-2α (white) with GFAP (magenta) in astrocytes of ON (arrows). (C) Longitudinal section of OHT ON shows an increase in HIF-1α level (white) compared with control. Colocalization of HIF-1α (white) with Iba-1 (magenta) in microglia of ON (arrows). (D) Longitudinal section of ON shows no difference in HIF-2α level (white) in OHT ON compared with control. Colocalization of HIF-2α (white) with Iba-1 (magenta) in astrocytes of ON (arrows). CTB (green) and DAPI (blue). n = 3 independent control ONs and 3 independent OHT ONs. Scale bar: 25 μm.

In ON, HIF-1α showed increased immunolabel with OHT compared with control (Figs. 6A, 6C). Both HIF-1α and HIF-2α colocalized with GFAP (Figs. 6A, 6B) and Iba-1-positive microglia (Figs. 6C, 6D) in the ON. The totality of the mRNA and protein findings for the HIFs, in addition to the pimonidazole labeling, indicate that the bead model–induced OHT drove hypoxia in the glia and RGCs 4 weeks after OHT but not at 2 weeks after OHT. In the ON, both astrocytes and microglia colocalized with HIF-1α and HIF-2α.

### Oxidative Stress Accumulation With Impaired Antioxidant Defense

**Figure 7:**
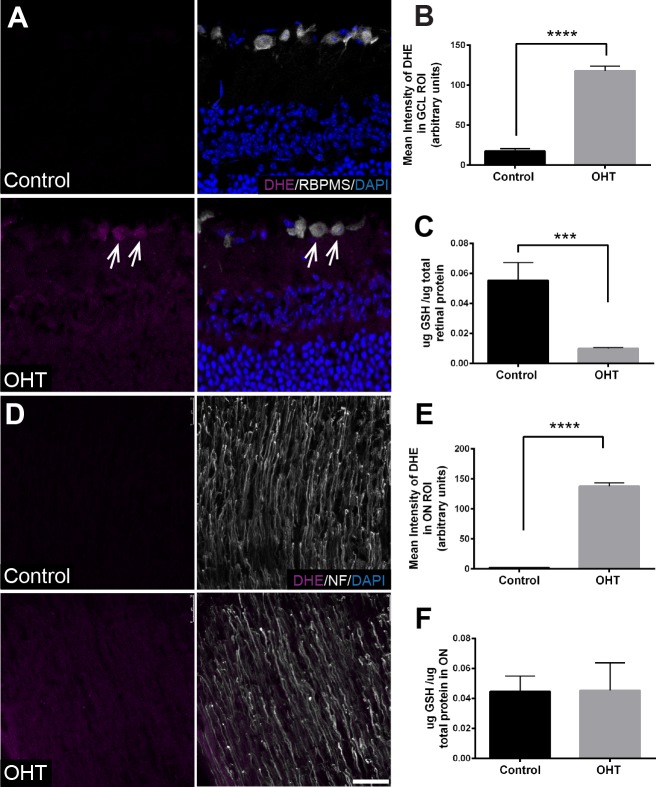
Superoxide accumulation in the retina and ON and decreased GSH in the retina after 4 weeks of OHT. (A) Immunofluorescence of sagittal retinal sections shows dihydroethidium (DHE, magenta), a marker of superoxide production, in OHT retina compared with control. Colocalization of DHE with RBPMS in RGCs of the OHT retina (arrows). RGCs immunolabeled with RBPMS (white), nuclei by DAPI (blue). Retinal layers labeled GCL, INL, and ONL. (B) Significant increase (****P < 0.0001) in the mean intensity of DHE in the GCL (118 ± 6.023, n = 12) of OHT retinas compared with control (17.52 ± 4.865, n = 8). Scale bar: 25 μm. (C) Significant decrease (***P < 0.001) of total GSH level in OHT retinas (0.010 ± 0.001, n = 7) compared with control (0.055 ± 0.012, n = 5). (D) Immunofluorescence of longitudinal ON sections show DHE accumulation as a result of OHT compared with control ONs; neurofilament immunolabel of axons in white. (E) The mean intensity of DHE in ONs was significantly greater (****P < 0.0001) in the OHT ONs (137.7 ± 5.668, n = 12) compared with control (1.79 ± 0.479, n = 8). (F) No significant difference (P > 0.05) in total GSH between control ONs (0.044 ± 0.010, n = 5) and OHT ON (0.045 ± 0.018, n = 8).

To show the magnitude of oxidative stress in retina and myelinated ON with increasing IOP, we measured superoxide levels and total reduced GSH in the retina and ON after 4 weeks of OHT. Superoxide levels as measured by DHE fluorescence showed significantly increased mean intensity of DHE in the GCL of OHT compared with control retina (Figs. 7A, 7B). In the ON of bead-injected mice, there was also significantly increased mean intensity of DHE fluorescence compared with control (Figs. 7D, 7E). Total GSH levels decreased significantly with OHT compared with control retina (Fig. 7C), whereas there was no change in GSH levels with OHT compared with control in ON (Fig. 7F).

**Figure 8:**
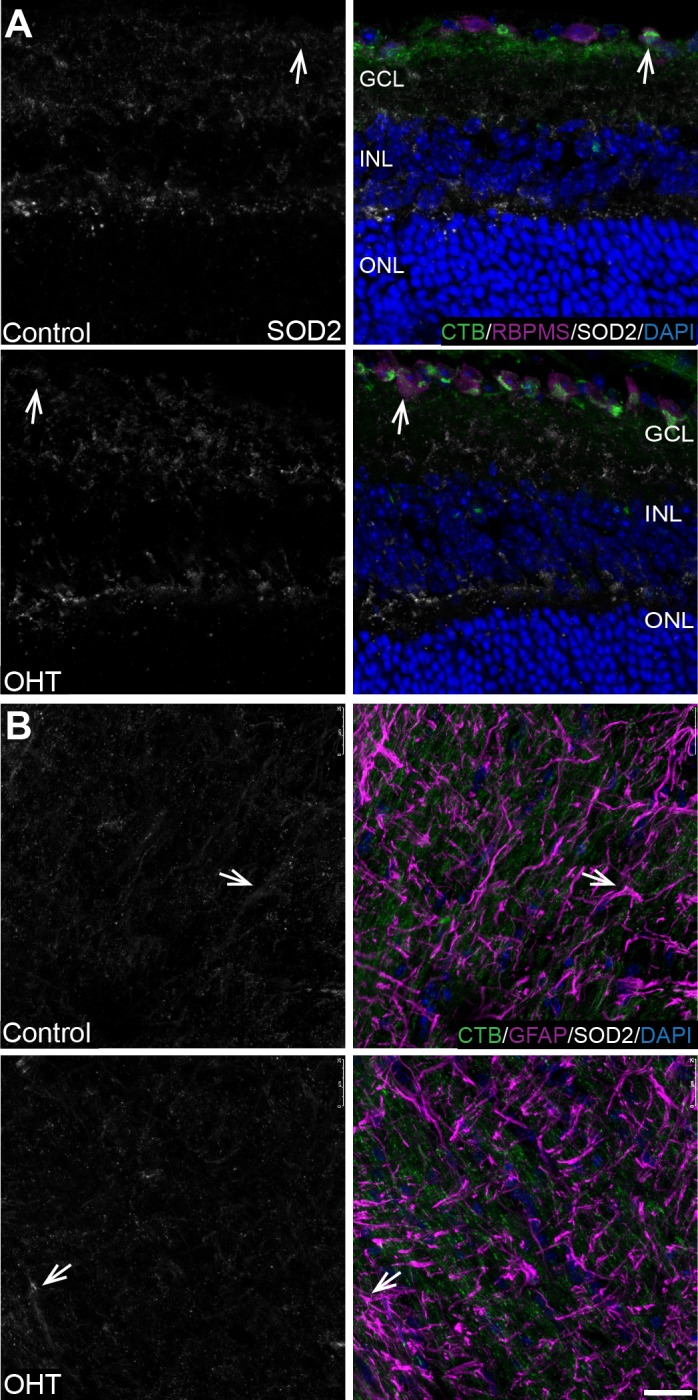
SOD2 immunolabeling unchanged in retina and ON after 4 weeks of OHT. (A) SOD2 colocalization with RBPMS in GCL and SOD2 immunolabeling in INL of OHT and control retina (arrows). n = 3 independent control and 3 independent OHT retinas. Scale bar: 25 μm. Region of interest analysis of SOD2 immunolabel intensity in GCL and IPL showed no difference with OHT versus control, see text for details. (B) SOD2 colocalized with GFAP in the OHT and control ON (arrows). n = 3 independent control and 3 independent OHT ONs. Capillary electrophoresis of SOD2 in ON showed no difference in protein levels between OHT and control, see text for details. Scale bar: 25 μm.

Immunofluorescence showed SOD2 immunolabel throughout the inner retina from OHT and control mice. SOD2 colocalization with RBPMS-positive RGCs in the GCL was observed in OHT and control retina (Fig. 8A). There was no significant difference (*P* > 0.05) in the mean intensity of SOD2 immunolabel in the region of interest selected for analysis of the GCL with OHT (56.26 ± 27.71, *n* = 3) and control (19 ± 2.485, *n* = 3); and analysis of the inner plexiform layer (IPL) with OHT (41.27 ± 10.62, *n* = 3) and control (48.14 ± 5.059, *n* = 3). In the ON, SOD2 colocalized with GFAP in both the OHT and control groups (Fig. 8B). There was no significant difference (*P* > 0.05) in SOD2 protein level in OHT retina and ON compared with control, as measured by capillary electrophoresis in the retina (OHT 0.176 ± 0.044, *n* = 7 vs. control 0.160 ± 0.052, *n* = 6) and ON (OHT 0.174 ± 0.066, *n* = 8 vs. control 0.165 ± 0.025, *n* = 6). Overall for oxidative stress analysis, the bead model–induced OHT triggered a significant accumulation of superoxide concomitant with an impaired GSH antioxidant system and no appreciable alteration in SOD2 immunolabel.

### P62 Expression and Autophagy Induction

**Figure 9:**
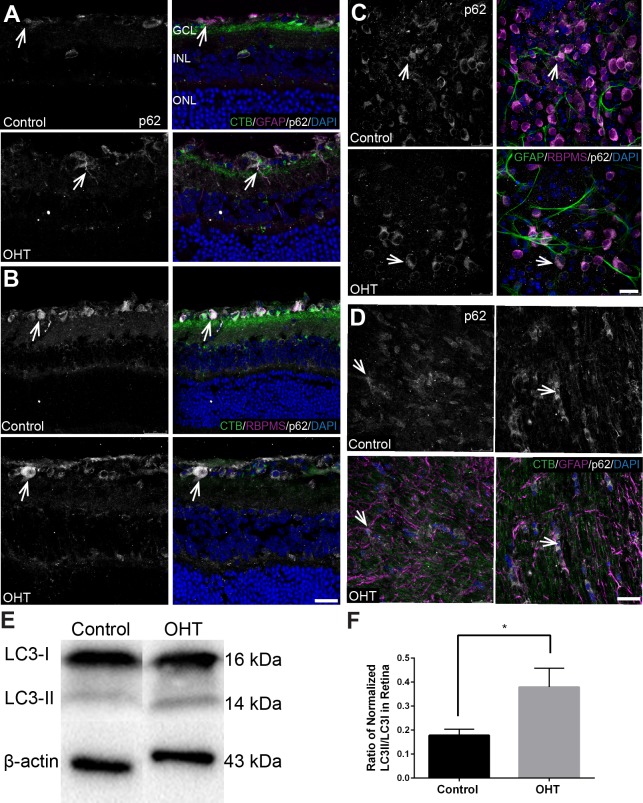
Autophagy induction with no significant change of p62 after 4 weeks of OHT. (A) Sagittal sections of retina show p62 (white) colocalization (arrows) with GFAP (magenta) in control and OHT retina. n = 4 independent control and 4 independent OHT retinas. (B) Sagittal sections of retina show colocalization of p62 (white) with RBPMS (magenta) in RGCs (arrows) of OHT and control. n = 4 independent control and 4 independent OHT retinas (C) Whole-mount retinas show colocalization of p62 (white) with RBPMS (magenta) in the cytoplasm of RGCs (arrows) of OHT and control. n = 3 independent control and 3 independent OHT retinas. (D) Longitudinal sections of ON show colocalization (arrows) of p62 (white) with GFAP (magenta) in control and OHT ON. Green label is CTB. n = 4 independent control and 4 independent OHT retinas. Scale bar: 25 μm. (E and F) Significant increase (*P < 0.05) of the normalized ratio of LC3II/LC3I in the retinas of OHT (0.379 ± 0.080, n = 7) compared with control (0.178 ± 0.025, n = 6).

To determine the level of p62 under hypoxia and superoxide accumulation, we conducted immunolabeling, transcript, and protein analysis of p62 in retina and ON of bead-injected compared with control mice. Immunolabeling showed colocalization of p62 in the astrocytes, Müller glia, and RGCs in the retina of both OHT and control groups (Fig. 9). There was no significant difference (*P* > 0.05) in the mean intensity of p62 immunolabel in the region of interest selected for analysis of the GCL with OHT (160 ± 13, *n* = 3) versus control (168.9 ± 7.8, *n* = 3); and in the ON, OHT (97.31 ± 5.7, *n* = 3) versus control (97.24 ± 6.6, *n* = 3). We observed no significant difference (*P* > 0.05) in *p62* transcripts in retina (OHT 0.097 ± 0.002, *n* = 4 vs. control 0.088 ± 0.005, *n* = 3) and ON (OHT 0.093 ± 0.004, *n* = 7 vs. control 0.095 ± 0.004, *n* = 3). We conducted capillary electrophoresis protein analysis of p62 and found a nonsignificant decrease (*P* > 0.05) of p62 levels with OHT in the retina (OHT 0.045 ± 0.016, *n* = 7 vs. control 0.063 ± 0.026, *n* = 6) and ON (OHT 0.056 ± 0.010, *n* = 8 vs. control 0.071 ± 0.027, *n* = 6). The ratio of LC3-II to LC3-I showed a significant increase in OHT compared with control retina (Figs. 9E, 9F). These data show that bead model–induced OHT resulted in a significant increase in autophagy induction with no significant change in p62 level and transcripts.

## Discussion

The OHT model used here triggered a hypoxic response in Müller glia, microglia, and astrocytes, as well as within RGCs of the retina after 4 weeks of IOP elevation, a unique observation. These results were confirmed by the detection of a significant increase of *Hif-1α* and *Hif-2α* transcripts and the consequent increase in *Glut-1* transcripts. This hypoxic response after 4 weeks of IOP elevation was accompanied by a significant increase in superoxide in the OHT retina and ON, and significantly decreased total GSH level and increased LC3 lipidation in the OHT retina. Interestingly, hypoxia was not detected at 2 weeks of OHT.

Previous research had shown that HIF-α gets activated during hypoxic conditions as a cellular adaptation to low oxygen, resulting in a reduction of electron transport chain activity in an effort to reduce ROS.^16^ Low oxygen tension can severely reduce cell energy availability with corresponding detrimental effects on the energy-demanding retina. Therefore, prolonged hypoxia and oxidative stress would be damaging to RGCs and contribute to glaucoma progression. It is worth noting that at early stages of hypoxia, induction of HIF-*α* could serve to protect cells from destruction. Zhu et al.^32^ previously showed the neuroprotective effect of intermittent hypoxia in the retina of an acute retinal ischemic model in rats; however, prolonged hypoxia can lead to cell death. Excessive HIF-α activation may be associated with the activation of the cell death program through p53, which has been suggested to be a transcriptional activator of neuronal apoptosis in glaucoma.^5^ This is consistent with our results, as we showed a significant increase in *Hif-1α* and *Hif-2α* transcripts concurrent with the degeneration of RGCs and axons and impaired anterograde axonal transport after 4 weeks of IOP elevation.

In the retina after 4 weeks of IOP elevation, total GSH was significantly decreased, but no difference in SOD2 level was observed despite a significant superoxide accumulation in OHT eyes compared with control. GSH level was similarly reduced in the chronic DBA/2J model of glaucoma.^33^ Hypoxia-induced reduction in GSH levels requires mitochondrial ROS (mtROS) such as superoxide.^34^ Significant decreases of GSH during hypoxia in rat brain^35^ indicate the retina responds similarly to the rest of the central nervous system. A superoxide increase without a concomitant increase in GSH with OHT suggests that the GSH may be used at a rate faster than it can be synthesized. Evidence for this interpretation includes a recent study that characterized the role of PGC-1α in the regulation of the modifying subunit (GCLM) of the GSH biosynthetic enzyme glutamate cysteine ligase.^36^ This group found that AMPK activation can increase PGC-1α and rescue GSH levels specifically in the astrocytes of a retina undergoing ischemia/reperfusion.^36^ However, we recently showed significant AMPK activation that accompanied PGC-1α downregulation in the ON of the chronic DBA/2J model of glaucoma.^35^,^37^ These mixed reports may be explained by the differences of models being used and the cell- and tissue-specific PGC-1α activity, as previously suggested.^36^ If PGC-1α is downregulated with bead model–induced OHT, then one might expect the significantly decreased GSH, as observed here.

Consistent with our findings of hypoxia induction and GSH reduction after 4 weeks of IOP elevation, we observed significant superoxide accumulation as measured by DHE. We observed no change in SOD2, which may have allowed superoxide to accumulate and reduce GSH levels. Our SOD2 immunolabeling shows greatest intensity in the GCL, IPL, and outer plexiform layer (OPL), but not the anticipated labeling of all retinal mitochondria,^38^ the location of SOD2. This suggests our antibody dilution constrained our view of SOD2; however, it is not anticipated that areas in which mitochondria are not concentrated would have made a significant difference to the observed outcome. Of particular interest was the greater intensity of DHE labeling in the RGCs of the bead-injected mouse eyes. SOD2 insufficiency could have contributed to the accumulation of DHE in the retina; however, we were using whole retinal protein for this analysis and potential differences across the retinal layers could obscure local changes.

After 4 weeks of IOP elevation, hypoxia was evident across retinal layers and cell types in the OHT retina but was especially prominent in glia via pimonidazole immunolabeling. Pimonidazole was injected before animals were killed, representing a snapshot of hypoxia 4 weeks after the commencement of IOP increase in these retinas. Significantly, we saw no pimonidazole labeling after 2 weeks of OHT, although significant loss of RGCs, axons, and anterograde transport was evident, and RGC loss increased further after 4 weeks of OHT. The lack of observed hypoxia at 2 weeks makes it less likely that hypoxia is driving RGC death. However, RGCs could experience hypoxia before or after either of the 2- or 4-week time points; additional time points could clarify. It also may be the case that RGCs and their axons in retina, ONH, and ON experienced earlier-onset hypoxia that was subsequently managed, thereby leaving no sign. This may be especially true at the onset of OHT.^3^

Chidlow et al.^3^ showed significant hypoxia in the ONH with 1 and 3 days of OHT, suggesting a very early wave of hypoxia that resolves, to be replaced by intermittent hypoxic episodes, at least 4 weeks after OHT commences. A separate study, using a rat model of OHT, did not detect hypoxia in microglia and neurons; however, that study also used a different timeline and IOP levels than those used here.^39^ As our study and the study by Chidlow et al.^3^ suggest, timing is critical to observations of hypoxia. Hypoxia was coincident with axon transport deficit at 1 and 3 days of OHT for Chidlow et al.^3^; this could have contributed to the scale of injury we observed at 2 weeks in our model of OHT (26% axon degeneration). By 4 weeks of OHT, we observed no additional axon degeneration in the ON, a result that corroborates recent findings of significant RGC changes (dendritic pruning and axon dysfunction) after 2 weeks of OHT that do not progress from 2 to 4 weeks.^40^ Future analysis of the very early post-IOP elevation period in our model of OHT may provide more insight into the role of hypoxia for axon degeneration.

In vitro studies have shown that glia (specifically astrocytes) are responsible for regulating GSH biosynthesis and release to protect neurons from oxidative stress.^41^ By examining hypoxia and GSH at 4 weeks after OHT, we may be observing that glia effectively assisted neurons in the resolution of oxidative stress but are not able to continue to manage long-term hypoxic injury. This would implicate glia in the ROS accumulation occurring through the retina and ON. Hypoxic astrocytes were evident in all retinas tested. Hypoxic RGCs, Müller glia, and microglia were evident in a subset of the retinas tested. Our findings also indicate that retinal Müller glia, microglia, and astrocytes are more prone to hypoxia as a result of IOP elevation than glia in the ON after 4 weeks of OHT. Glia of the ONH and ON did not show hypoxia at 2 or 4 weeks of IOP elevation. There was no relationship that could be determined between IOP and pattern or intensity of pimonidazole labeling in the retina after OHT. Any variability in RGC number or axon number also did not correspond to differences in hypoxia as determined by pimonidazole.

Consistent evidence of hypoxia in the astrocytes, as we observed here, may indicate a specific issue with the inner retinal vasculature. Valiente-Soriano et al.^42^ examined inner retinal vasculature after 2 weeks of raised IOP by laser photocoagulation in rats. A direct comparison of inner retina vasculature from areas with intact retrogradely labeled RGCs and those without RGCs did not differ, suggesting little contribution of the vasculature to RGC survival.^42^ We did not evaluate the vasculature in the present study; however, a future investigation of vasculature (choroidal and inner retina) after 2 and 4 weeks of OHT could be informative.

After 4 weeks of IOP elevation, we show a significant increase in *Glut-1* transcripts in the OHT retina along with a significant increase in *Hif-1α* and *Hif-2α*. The increase in *Glut-1* transcripts is expected, as the deprivation of O_2_ (hypoxia) can shift cell metabolic activity toward glycolysis. In the retina and ON, GLUT1 is primarily expressed in glial cells. We observe no change in *Glut1* transcripts in the bead-injected mouse ON despite a significant increase in *Hif-1α* in ON. *Glut1* mRNA also is not changed in 10-month-old DBA/2J glaucomatous ON, despite significant decrease in GLUT1 protein.^43^ Glucose has a critical function in maintaining cellular GSH levels under normoxic and hypoxic conditions by providing nicotinamide adenine dinucleotide phosphate through the pentose phosphate pathway.^44^ Oxygen glucose deprivation, unlike hypoxia alone, triggered oxidative stress and mitochondrial dysfunction in primary cultured neurons, indicating that glucose metabolism could play a critical role in neuroprotection during hypoxia.^45^ Using an oxygen-induced retinopathy mouse model, Mowat et al.^27^ showed that HIF-1α staining was prominent in cells across the INL and GCL, whereas HIF-2α was highly restricted to Müller glia and astrocytes. In addition, they showed that HIF-2α is expressed at a higher level than HIF-1α within the hypoxic inner retina. Our findings in OHT were consistent with that study, and showed activation of both HIF-1α and HIF-2α with retinal hypoxia and IOP elevation in RGCs and Müller glia, astrocytes, and microglia.

In the ON, total GSH and SOD2 levels in OHT were not different from control despite the detection of significant increases of superoxide by DHE. In accordance, hypoxia, as assayed by pimonidazole immunolabel, was largely absent in the ON and ONH with comparably low labeling in control and the bead glaucoma model after 2 weeks and 4 weeks of IOP elevation; however, there was a significant increase in *Hif-1α* transcript after 4 weeks of OHT. We do not know the threshold necessary for *Hif-1α* induction and how it compares with the threshold required for pimonidazole adduct labeling, although these data suggest adduct creation may occur at a higher threshold. Interestingly, we found that superoxide accumulation was concomitant with hypoxia increase in the retina but not the ON. Mitochondrial ROS (specifically those generated at mitochondrial Complex III, such as superoxide) are essential to stabilize *Hif-α* during hypoxia.^37^,^46^ It was previously shown that acute hypoxia produces a superoxide burst in cells.^47^ ROS, including superoxide, may be stabilizing *Hif-1α* in the ON to a degree that is sub-threshold for pimonidazole-positive hypoxia while detectable using DHE.

Rantanen et al.^48^ showed that p62 is required to trap PHD3 in aggregates and block its interaction with HIF-1α. They found that downregulation of p62 accompanied the adjustment of cellular energy metabolism to meet the diminished oxygen availability in hypoxic carcinoma cells. A nonsignificant decrease of p62 protein level was detected in OHT retina and ON compared with control. The lack of the expected decrease in p62 may be traced to p62 involvement in many pathways within the cell, rendering p62 unavailable to regulate the interaction of PHD3 and HIF-1α, which may partially explain the upregulation of *Hif-1α* transcripts that were detected in this study. Alternatively, the adjustment of energy metabolism could have occurred before the time point at which we assayed p62. Recently, it has been shown that oxidation-dependent oligomerization of p62 promotes autophagy and is a pro-survival mechanism.^49^ Oxidation of p62 might be triggered by specific ROS such as the H_2_O_2_ shown in that study. The significant increase in the ratio of LC3-II to LC3-I shown here indicates an increase in LC3 lipidation triggered by IOP elevation and hypoxia with the bead OHT model. Autophagy involvement in glaucoma pathology is still to be determined. Autophagy has been associated with neuroprotection^50^; however, excessive autophagy induction can lead to RGC degeneration. Park et al.^51^ showed that the inhibition of autophagy is protective in a rat model of glaucoma. We have previously shown LC3 lipidation, an increase in autophagosomes, and a decrease in lysosomes in the chronic DBA/2J model of glaucoma, altogether suggesting disrupted autophagy.^28^,^31^

Microglia continuously respond to alterations in cellular homeostasis resulting from hypoxia, oxidative stress, and other stimuli. We observed hypoxic microglia in retina and ON, consistent with their activation in glaucoma, as shown in previous reports.^52^–^54^ Prolonged hypoxia may not only directly damage neurons but may promote neuronal injury indirectly via activation of microglial cells.^52^–^54^ Activated microglia can initiate neuronal and oligodendrocyte injury and exacerbate a variety of neurological conditions by secreting proinflammatory molecules, including nitric oxide, TNF-α, and IL-1.^53^ These cytokines have long been implicated in the pathogenesis of glaucoma.^55^ Sivakumar et al.^56^ showed that activated microglia in hypoxic neonatal retina produce increased amounts of inflammatory cytokines that could induce RGC death. Future studies are warranted to investigate the crosstalk between prolonged hypoxia and inflammation in glaucoma.

Our findings characterize, for the first time, the incidence of hypoxia, hypoxia-related transcripts, oxidative stress, and autophagy 2 and 4 weeks after OHT. We show IOP-related induction of hypoxia in the retina with concomitant superoxide accumulation, GSH depletion, and an increase in LC3 lipidation, suggesting that hypoxic signaling in response to IOP elevation 4 weeks after OHT contributes to the progression of neuronal damage in glaucoma.

## Supplementary Material

Supplement 1
