# Supplementary material for: Evidence of Hypoxic Glial Cells in a Model of Ocular Hypertension
Source: Invest Ophthalmol Vis Sci. 2019 Jan;60(1):1–15. doi: 10.1167/iovs.18-24977 (PMC6322635; doi:10.1167/iovs.18-24977)
Supplement: Supplement 1 [file iovs-59-15-22_s01.pdf]

Supplementary Table 1. Numbers shown represent number of samples used for each experiment. For the CTB anterograde transport (percent area fraction) in the superior colliculus (SC) determination at 2 weeks, n=8 SCs with OHT and n=12 Ctrl SCs; at 4 weeks, n=10 SCs with OHT and control. Ctrl: control, OHT: ocular hypertension, NA: not applicable. NT: not tested.

**Experiment**	**Eyes/Retinas**	**ONs**
IOP Measurements	Ctrl (4weeks): 29<br>OHT(4 weeks): 54<br>Ctrl (2 weeks): 16<br>OHT(2 weeks): 17	NA
% RGC Loss	Ctrl: 20<br>OHT (2 weeks): 11<br>OHT (4 weeks): 25	NA
% Axon Loss	NA	Ctrl: 12<br>OHT (2 weeks): 9<br>OHT (4 weeks): 12
Hypoxia (Pimonidazole-Positive Cells) Detection	Ctrl (4 weeks): 8<br>OHT (4 weeks): 10<br>Ctrl (2 weeks): 4<br>OHT (2 weeks): 8	Ctrl (4 weeks): 8<br>OHT (4 weeks): 10<br>Ctrl (2 weeks): 4<br>OHT (2 weeks): 8
qPCR	Ctrl: 3<br>OHT (4 weeks): 4	Ctrl: 3<br>OHT (4 weeks): 7
IHC: Hif-1α and Hif-2α with GFAP and Iba-1 in sections	Ctrl: 3<br>OHT (4 weeks): 3	Ctrl: 3<br>OHT (4 weeks): 3
IHC:<br>Hif-1α and RBPMS in whole mount retinas	Ctrl:4<br>OHT (4 weeks):6	NA
DHE Analysis	Ctrl: 8<br>OHT (4 weeks): 12	Ctrl: 8<br>OHT (4 weeks): 12
GSH Analysis	Ctrl: 5<br>OHT (4 weeks): 7	Ctrl: 5<br>OHT (4 weeks): 8
IHC of SOD2 Immunolabeling analysis	Ctrl: 3<br>OHT (4 weeks): 3	Ctrl: 3<br>OHT (4 weeks): 3
Protein Analysis of SOD2 (WES)	Ctrl: 6<br>OHT (4 weeks): 7	Ctrl: 6<br>OHT (4 weeks): 8
IHC: p62 with GFAP and RBPMS in sections	Ctrl: 4<br>OHT (4 weeks): 4	Ctrl: 4<br>OHT (4 weeks): 4
P62 Immunolabeling Analysis	Ctrl:3<br>OHT (4 weeks): 3	Ctrl:3<br>OHT (4 weeks):3
IHC:<br>P62, RBPMS and GFAP in whole mount retinas	Ctrl:3<br>OHT (4 weeks): 3	NA
Protein Analysis of p62 (WES)	Ctrl: 6<br>OHT (4 weeks): 7	Ctrl: 6<br>OHT (4 weeks): 8
Protein Analysis of LC3II/LC3I (WB)	Ctrl: 6<br>OHT (4 weeks): 7	NT
Protein Analysis of Hif-1α (WB)	Ctrl: 4	NT

	OHT (4 weeks): 6	
Protein Analysis of Hif-2α (WB)	Ctrl: 4<br>OHT (4 weeks): 6	NT
Protein Analysis of Hif-1α (WB)	Ctrl: 4<br>OHT (2 weeks): 4	NT
Protein Analysis of Hif-2α (WB)	Ctrl: 3<br>OHT (2 weeks) : 4	NT
